# A Male Patient Presents With Isolated Abducens Nerve Palsy: An Atypical Presentation of Ocular Myasthenia Gravis

**DOI:** 10.7759/cureus.57501

**Published:** 2024-04-03

**Authors:** Jaron C Sanchez, Markeeta T Belmar, Jason Sanchez, Joseph L Mercen, Jose Prieto

**Affiliations:** 1 Dr. Kiran C. Patel College of Osteopathic Medicine, Nova Southeastern University, Clearwater, USA; 2 School of Osteopathic Medicine, University of the Incarnate Word, San Antonio, USA; 3 Internal Medicine, Bond Clinic, P.A., Winter Haven, USA

**Keywords:** isolated cranial nerve palsy, acetyl-cholinesterase inhibitors, autoimmune neuromuscular disease, sixth cranial nerve palsy, isolated abducens nerve palsy, ocular myasthenia gravis, myasthenia gravis (mg)

## Abstract

Myasthenia gravis is an autoimmune disease of the neuromuscular junction caused by autoantibodies directed against the acetylcholine receptors. It presents with skeletal muscle weakness, often initially presenting with ocular symptoms such as ptosis and diplopia. When myasthenia gravis is isolated to only ocular symptoms, it is referred to as ocular myasthenia gravis (OMG). Here, we present an atypical initial presentation of OMG in a 68-year-old male patient presenting with isolated abducens nerve palsy at the initial onset. With this case report, we highlight the importance of a thorough history and clinical assessment necessary for a timely diagnosis of OMG in patients who present with isolated abducens nerve palsy.

## Introduction

Myasthenia gravis (MG) is an autoimmune disease of the neuromuscular junction caused by autoantibodies directed against the acetylcholine receptors (AchR). It affects striated muscles leading to characteristic fluctuating fatigable weakness of the ocular, bulbar, respiratory, axial, and extremity muscles [1]. MG at initial onset usually manifests first with ocular symptoms presenting typically with ptosis and/or diplopia. Moreover, when MG is isolated to only ocular symptoms, it is referred to as ocular myasthenia gravis (OMG), which tends to affect older patients greater than 60 years old. Patients can also atypically present with isolated cranial nerve palsies such as abducens nerve palsy characterized by weakness in only the lateral rectus muscle. Isolated cranial nerve palsies in most patients are likely to have vascular etiology, but it can also be caused by trauma or mass effect by a brain tumor [2,3]. As OMG can mimic these cranial nerve palsies, it is important to first rule out vascular and other etiologies [4]. However, in the case that these etiologies are negative, clinicians should have high suspicion for OMG. Our case report looks into an atypical presentation of OMG in a 68-year-old male who initially presents with isolated, unilateral abducens nerve palsy. This case highlights the importance of a thorough history and clinical assessment necessary for a timely diagnosis of OMG.

## Case presentation

We report a case of a 68-year-old male who presented with double vision in his right eye with associated photophobia for two weeks. He reported that “he woke up one morning with it.” He has a known underlying stable history of aortic stenosis, pulmonary hypertension, insomnia, and prostate cancer. He denies a family medical history of diabetes, hypertension, cardiovascular, thyroid, or autoimmune disease. Active medications include fluticasone 50 mcg nasal spray suspension PRN for allergies and eszopiclone 3 mg tablet PRN for insomnia.

**Figure 1:**
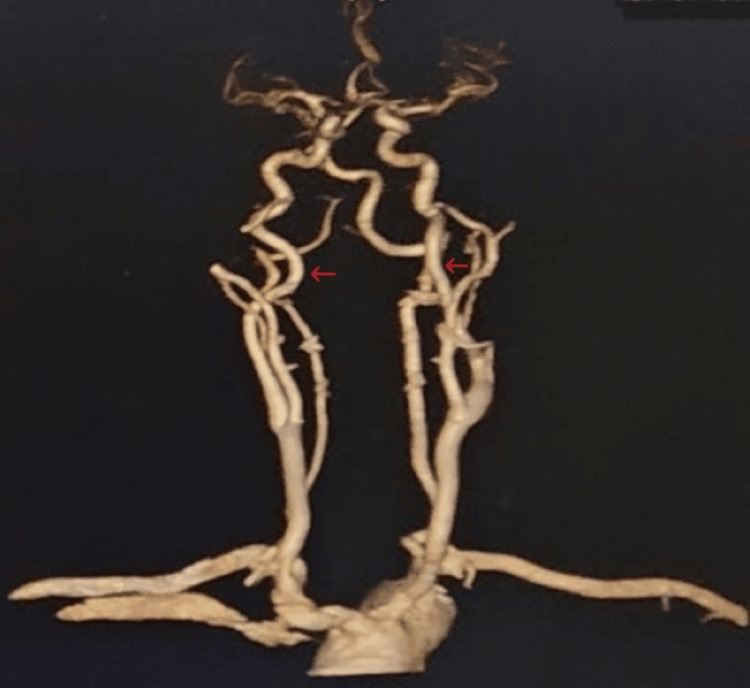
Three-dimensional reconstruction of head and neck CTA. No evidence of internal carotid artery stenosis was found. CTA: computed tomography angiogram.

Outpatient comprehensive ophthalmology diagnosed him with sixth cranial nerve palsy and was sent to the hospital for an MRI of the head and a CT angiogram of the head and neck. The physical examination was significant for right eye medial deviation with double vision and no acute changes in sensory or motor function or focal neurological deficits (Table 1). Vital signs and labs were unremarkable (Table 2). MRI of the head showed cerebellar tonsillar ectopia and no acute intracranial abnormalities. CT scan of the head and neck did not show internal carotid artery stenosis or large vessel occlusion, vascular malformation, or aneurysm in the brain (Figure 1). The patient refused to wait for an inpatient neurology evaluation but followed up with neuro-ophthalmology soon after the hospital discharge.

**Table 1:** Physical examination findings during the hospital visit. HENT: head, ears, nose, throat.

Physical Examination	Negative or Positive	Findings
Constitutional	Negative	Active, alert, oriented. No acute distress.
HENT	Negative	Normocephalic. Normal hearing, oral. Mucosa moist. No pharyngeal erythema. No sinus tenderness.
Eyes	Positive	Medial deviation of the right eye. Normal conjunctiva.
Neck	Negative	Supple, non-tender. No carotid bruit. No jugular venous distension. No lymphadenopathy.
Respiratory	Negative	Lungs are clear to auscultation. Respirations non-labored. Breath sounds equal. Symmetrical chest wall expansion.
Cardiovascular	Negative	Normal rate, regular rhythm. No murmur. Good pulses equal in all extremities. No edema.
Gastrointestinal	Negative	Soft, non-tender, non-distended. Normal bowel sounds. No organomegaly.
Genitourinary	Negative	No costovertebral angle tenderness. No inguinal tenderness.
Lymphatics	Negative	No lymphadenopathy neck, axilla, and groin.
Musculoskeletal	Negative	Normal range of motion. No acute change in strength and gait. No tenderness. No swelling.
Neurologic	Negative	No acute change in motor or sensory function. No focal deficits.
Skin	Negative	No rash. No cyanosis.
Psychiatric	Negative	Cooperative, appropriate mood and affect. No suicidal ideas.

**Table 2:** Lab findings during the hospital visit. CBC: complete blood count; WBC: white blood cells; Hgb: hemoglobin; HCT: hematocrit; PLT: platelets; BMP: basic metabolic panel; Na+: sodium; K+: potassium; Ch-: chloride; CO_2_: carbon dioxide serum; BUN: blood urea nitrogen; BUN/Cr: blood urea nitrogen-to-creatinine ratio; Ca^2+^: calcium serum; ALT: alanine aminotransferase; AST: aspartate aminotransferase; Mg^2+^: magnesium; eGFR: estimated glomerular filtration rate; AKI: acute kidney injury; RBC: red blood cells; MCV: mean corpuscular volume; MCH: mean corpuscular hemoglobin; MCHC: mean corpuscular hemoglobin concentration; RDW: red blood cell distribution width; MPV: mean platelet volume.

Labs	Result	Reference Interval
CBC		
WBC	6.1	4-10 x 10^9^/L
Hgb	13.7	13-17 g/dL
HCT	38.5 (L)	40-52%
PLT	177	150-400 x 10^9^/L
BMP		
Na+	140	135-145 mmol/L
K+	4.2	3.5-5 mmol/L
Ch-	108 (H)	95-105 mmol/L
CO_2_	25	23-29 mmol/L
Glucose	117 (H)	65-110 mg/dL
BUN	14	8-21 mg/dL
Creatinine	0.849	0.8-1.3 mg/dL
General Chemistry		
BUN/Cr	16	10-20 mg/dL
Ca^2+^	8.8	8.4-10.2 mg/dL
Total protein	6.3	6.0-8.0 g/dL
Albumin	4.0	3.5-5.5 g/dL
Total bilirubin	0.5	0.1-1.0 mg/dL
Alkaline phosphatase	93	50-100 U/L
ALT	16	5-30 U/L
AST	17	5-30 U/L
Anion gap	7	4-12 mmol/L
Mg^2+^	2.1	1.7-2.2 mg/dL
eGFR	>60	>60 mL/min/1.73 m^2^
AKI suspected	None	None
Hematology		
RBC	4.17 (L)	4.54-5.78 x 10^12^/L
MCV	92.4	80-100 fL
MCH	32.8 (H)	27.1-32.5 pg
MCHC	35.5	32.5-36.7 g/dL
RDW	13.9	1.5-14.5%
MPV	9.1 (H)	6.1-8.9 fL
Neutrophils, segmented	55.3	54-62%
Lymphocytes	24.9	14.1-45.8%
Monocytes	14.7 (H)	3-7%
Eosinophils	3.7	0.3-6.2%
Basophils	1.4 (H)	0.3-1.3%

During the neuro-ophthalmology visit, the patient denied any past ocular medical or surgical history. On examination, visual acuity in both eyes with correction was 20/25 (Table 3). Pupils in both eyes were equal, round, and reactive to light and accommodation. Intraocular pressure in both eyes was 18 mmHg (reference interval: 10-20 mmHg). Right lateral rectus deficit was noted during the extraocular movement (EOM) examination. Muscle balance also demonstrated increasing esotropia on right gaze. Dermatochalasis was noted in both eyes on the external exam. The rest of the external, slit lamp, lacrimal, and fundus exams were unremarkable. MG was suspected, and AchR antibodies were measured to rule it out. AchR binding antibody was positive at 1.86 nmol/L (reference interval: 0.00-0.24 nmol/L) and AchR blocking antibody was positive at 35% (reference interval: 0-25%). Based on the positive AchR antibodies in addition to isolated ocular manifestations, the patient was diagnosed with OMG. Moreover, a follow-up visit with outpatient neurology established no evidence of generalized MG (GMG) based on unremarkable findings in the physical examination. The physical examination demonstrated no appendicular or generalized weakness, no ptosis, normal respiratory effort, normal gait and balance, and normal coordination. Initial treatment with pyridostigmine 60 mg tablet three times daily was prescribed.

**Table 3:** Eye exam and labs from neuro-ophthalmology visit. OU: both eyes; IOP: intraocular pressure; APD: afferent pupillary defect; ERLA: equal reactive to light and accommodation; EOM: extraocular movement; OD: right eye; OS: left eye; MRD: margin to reflex distance.

Examination	Result	Reference Interval
Visual acuity w/ correction OU	20/25	-
IOP OU	18 mmHg	10-21 mmHg
Pupils OU		
APD	No	-
ERLA	Yes	-
Reaction	4+	4+
EOM		
OD	Lateral rectus deficit	-
OS	EOMI	-
Muscle balance	Esotropia increasing on the right gaze	-
External Exam		
Nystagmus	No	-
MRD OU	2	4.0-5.0 mm
Hertel	Symmetric w/ no proptosis	-
Levator function OU	15	13-17 mm
Dermatochalisis upper lid OU	Present	-
Dematochalisis lower lid OU	Present	-
Lid crease	Normal	-
Ectropion upper lid OU	None	-
Ectropion lower lid OU	None	-
Entropion upper lid OU	None	-
Entropion lower lid OU	None	-
Lagophthalomos OU	None	-
Eyelid retraction upper lid OU	None	-
Eyelid retraction lower lid OU	None	-
Hyperophthalmia	None	-
Lacerations upper lid OU	None	-
Lacerations lower Lid OU	None	-
Masses upper lid OU	None	-
Masses lower lid OU	None	-
Lacrimal gland OU	None	-
Slit lamp exam		
Anterior segment external OU	Clear	-
Conjunctiva OU	White	-
Sclera OU	White	-
Cornea OU	Clear	-
Anterior chamber OU	Deep and quiet	-
Iris OU	Pupil round and regular	-
Lens OU	Clear	-
Anterior vitreous OU	Clear	-
Lacrimal Exam		
Puncta upper lid OU	Open in normal position	-
Puncta lower lid OU	Open in normal position	-
Discharge OU	None	-
Probe and Irrigation OU	Open	-
Fundus Exam		
Vitreous OU	Clear	-
Cup:disc ratio OU	0.2	<0.7
Optic disc OU	No edema, no vascularization, good color	-
Vessels OU	WNL	-
Macula OU	Good foveal reflex, no drusens	-
Periphery OU	Attached with no breaks or tears	-
	20D & 90D ophthalmoscopic exam - normal	-
Titers		
Acetylcholine receptor binding antibody	1.86 nmol/L	0.00-0.24 nmol/L
Acetylcholine receptor blocking antibody	35%	0-25%

Two months later, the patient reported improvement in his vision, but the physical examination still demonstrated medial deviation of the right eye. The rest of the physical examination remained unremarkable with no evidence of GMG. The patient will continue to be monitored for any worsening of symptoms.

## Discussion

MG is an autoimmune disease of the neuromuscular junction caused by autoantibodies directed against the AchRs. It can occur at any age but typically has a bimodal distribution with younger patients affected being females and older patients affected being males [5]. MG affects striated muscles leading to characteristic fluctuating fatigable weakness of the ocular, bulbar, respiratory, axial, and extremity muscles [1]. It usually manifests first with ocular symptoms affecting extraocular muscles (e.g. superior rectus, inferior rectus, medial rectus, lateral rectus, inferior oblique, and superior oblique) and eyelid muscles (e.g. levator palpebrae superioris, superior tarsal muscle, and orbicularis oculi) in approximately 60% of patients. Therefore, at the initial onset, patients typically present with ptosis and/or diplopia. Ptosis is often unilateral or asymmetric on presentation and can alternate from one side to the other. Diplopia commonly involves the medial rectus, and the pupils are spared with no pupil abnormalities [6]. When MG is isolated to only ocular symptoms, it is referred to as OMG and predominantly affects male individuals.

In addition to ptosis and diplopia, OMG can also manifest atypically by mimicking isolated cranial nerve palsies. OMG can have isolated involvement of the inferior rectus and levator palpebrae superioris mimicking a pseudo-oculomotor nerve palsy, the lateral rectus mimicking an abducens nerve palsy, and the superior oblique mimicking a trochlear nerve palsy [7]. Rarely does MG initially present with glossopharyngeal and vagus nerve involvement which would present with dysphagia and dysphonia [8]. To our knowledge, there are limited studies or case reports that explore the prevalence of the different isolated cranial nerve palsies in patients with MG. Cleary et al. briefly touched on this in which EOMs were examined in 49 patients with OMG and determined that 72% had bilateral EOM involvement and that 4% presented with isolated abducens nerve palsy [2].

Although isolated abducens nerve palsy in patients with OMG is rare, abducens nerve palsy is the most common cranial nerve neuropathy in adults aged 60-70 years [9]. It is more likely to have a vascular etiology such as diabetes or hypertension, but it can also be caused by trauma or mass effect by a brain tumor [2,3]. Interestingly, there are also rare cases of vasculitic diseases such as giant cell arteritis that can present with abducens nerve palsy [10]. Oculomotor nerve palsy is commonly seen in patients with a supraclinoid aneurysm, vascular disease, and trauma, while trochlear nerve palsy is commonly seen in patients with frontal head trauma and vascular diseases [3]. As OMG can mimic these cranial nerve palsies, it is important to first rule out strokes and tumors in patients who present with cranial nerve palsies [4]. However, in the case that strokes and tumors are negative, clinicians should have a high suspicion of OMG and include it in their list of differential diagnoses. Therefore, our case highlights the importance of a thorough history and clinical assessment for a timely diagnosis of OMG.

Of the patients who present with OMG, two-thirds of these patients will develop signs and symptoms of extremity and bulbar muscle weakness classified as GMG within two years, while one-third will continue to have pure OMG. Studies have shown that patients with positive AchR antibodies, positive single-fiber electromyography, older age, female sex, or bilateral ptosis were associated with an increased risk of GMG conversion [11,12]. Treatment for OMG is generally treated with acetylcholine esterase inhibitors, but depending on the severity adding low-dose prednisone may be warranted. For treatment of GMG, long-term immunosuppressants are used [13]. Additionally, chronic low-dose prednisone used with oral immunomodulators has been shown to be moderately effective in delaying the progression of OMG to GMG [14]. Our patient has some of these risk factors such as older age with positive AchR antibodies in addition to taking only acetylcholine esterase inhibitors without additional immunosuppressants putting our patient at a higher risk for GMG conversion. To this end, this case emphasizes closer surveillance of patients with OMG.

## Conclusions

Isolated abducens nerve palsies in patients with OMG are rare. However, there are a few studies that explore the prevalence of the different isolated cranial nerve palsies which warrant further investigation. Moreover, when a patient is presented with isolated cranial nerve palsies, etiologies such as vascular diseases, trauma, and tumors should be ruled out first. Otherwise, there should be a high index of suspicion for OMG and included in the list of differential diagnoses. Furthermore, this case highlights the importance of a thorough history and clinical assessment necessary for a timely diagnosis of OMG in patients who present with isolated abducens nerve palsy. Additionally, there should be closer surveillance of patients with OMG due to increasing risk factors such as older age and positive AchR antibodies.
